# Age-Dependent Reduction in Neutralization against Alpha and Beta Variants of BNT162b2 SARS-CoV-2 Vaccine-Induced Immunity

**DOI:** 10.1128/Spectrum.00561-21

**Published:** 2021-12-01

**Authors:** Hitoshi Kawasuji, Yoshitomo Morinaga, Hideki Tani, Yumiko Saga, Makito Kaneda, Yushi Murai, Akitoshi Ueno, Yuki Miyajima, Yasutaka Fukui, Kentaro Nagaoka, Chikako Ono, Yoshiharu Matsuura, Hideki Niimi, Yoshihiro Yamamoto

**Affiliations:** a Department of Clinical Infectious Diseases, Toyama University Graduate School of Medicine and Pharmaceutical Sciences, Toyama, Japan; b Department of Microbiology, Toyama University Graduate School of Medicine and Pharmaceutical Sciences, Toyama, Japan; c Clinical and Research Center for Infectious Diseases, Toyama University Hospital, Toyama, Japan; d Department of Virology, Toyama Institute of Health, Toyama, Japan; e Laboratory of Virus Control, Center for Infectious Disease Education and Research (CiDER), Osaka University, Osaka, Japan; f Laboratory of Virus Control, Research Institute for Microbial Diseases (RIMD), Osaka University, Osaka, Japan; g Department of Clinical Laboratory and Molecular Pathology, Toyama University Graduate School of Medicine and Pharmaceutical Sciences, Toyama, Japan; University of Cincinnati

**Keywords:** neutralizing antibodies, receptor-binding domain, variants, SARS-CoV-2, BNT162b2

## Abstract

Vaccines against severe acute respiratory syndrome coronavirus-2 have been introduced. To investigate the relationship between vaccine-induced humoral immunity and patient age, we measured antibody levels and neutralization in vaccinated sera. Sera from 13 to 17 days after the second dose of the BNT162b2 vaccine were collected from health care workers at the University of Toyama (*n* = 740). Antibody levels were measured by the anti-receptor binding domain antibody test (anti-RBD test), and neutralization against wild-type (WT), α- and β-variant pseudotyped viruses were assayed using a high-throughput chemiluminescent reduction neutralizing test (htCRNT; positivity cutoff, 50% neutralization at serum dilution 1:100). Basic clinical characteristics were obtained from questionnaires. Antibodies were confirmed in all participants in both the anti-RBD test (median, 2,112 U/ml; interquartile range [IQR], 1,275 to 3,390 U/ml) and the htCRNT against WT (median % inhibition, >99.9; IQR, >99.9 to >99.9). For randomly selected sera (*n* = 61), 100.0% had positive htCRNT values against the α- and β-derived variants. Among those who answered the questionnaire (*n* = 237), the values of the anti-RBD test were negatively correlated with age in females (*P* < 0.01). An age-dependent decline in neutralization was observed against the variants but not against the wild-type virus (wild type, *P* = 0.09; α, *P* < 0.01; β, *P* < 0.01). The neutralizing activity induced by BNT162b2 was obtained not only against the wild-type virus, but also against the variants; however, there was an age-dependent decrease in the latter. Age-related heterogeneity of vaccine-acquired immunity is a concern in preventive strategies in the era dominated by variants.

**IMPORTANCE** Since mRNA vaccines utilize wild-type SARS-CoV-2 spike protein as an antigen, there are potential concerns about acquiring immunity to variants of this virus. The neutralizing activity in BNT162b2-vaccinated individuals was higher against the wild-type virus than against its variants; this effect was more apparent in older age groups. This finding suggests that one of the weaknesses of the mRNA vaccine is the high risk of variant infection in the elderly population. Because the elderly are at a higher risk of SARS-CoV-2 infection, the age-dependent decline of neutralization against viral variants should be considered while planning vaccination programs that include boosters.

## INTRODUCTION

Since the emergence of severe acute respiratory syndrome coronavirus-2 (SARS-CoV-2) in China, the COVID-19 pandemic has strongly limited our healthy lifestyles and economic activity. There are still no therapeutic breakthroughs, but vaccination is expected to be a specific and effective defense strategy for fighting COVID-19. Several vaccines against SARS-CoV-2 have been introduced and as of this date, vaccines manufactured by Pfizer (BNT162b2) have been administered in Japan. This vaccine was shown to have preventive effects against COVID-19 in 95% of individuals (1); however, SARS-CoV-2 variants have emerged since this report. Although it is well known that elderly are at a higher risk for COVID-19 (2), antibody response to the vaccine generally decreases with patient age (3) and similar findings have also been reported for BNT162b2 (4, 5). The efficacy of vaccine-induced immunity on the variants and the relationship between vaccine effectiveness and the age of the vaccinee are not fully understood.

The B.1.1.7 (α) variant, which originated in the United Kingdom, has a mutation, N501Y, which is related to an increased affinity of the virus to the angiotensin-converting enzyme 2 (ACE2) receptor expressed on targeted cells (6). The B.1.351 (β) variant, which originated in South Africa, has the mutations K417N, E484K, and N501Y, located in the receptor binding domain (RBD) of the spike (S) protein, which are related to reduced neutralization (7). The effectiveness of the Pfizer vaccine against these emerging variants has been reported to be similar to its efficacy against wild-type SARS-CoV-2 (8). In contrast, a reduction in the neutralization potential of vaccinated sera against variants has also been reported (9–11). Therefore, a continuous evaluation of vaccine efficacy is required.

Neutralizing antibodies have protective functions against pathogens. We previously established the chemiluminescence reduction neutralization test (CRNT) for evaluating neutralizing activity against SARS-CoV-2 by using pseudotyped virus (12). The high-throughput CRNT (htCRNT), which is a modified method using 384-well microplates, also has a good correlation with the CRNT (12). These methods assess the inhibition of infectivity in target cells. In contrast, it is impossible to assess protective function directly by *in vitro* diagnostic (IVD) antibody tests because they do not assess inhibition against viral infection. However, some tests can provide quantitative values of antibodies and help us to speculate on the state of acquired immunity. In this study, we confirmed that the Elecsys Anti-SARS-CoV-2 S immunoassay, which is an anti-RBD antibody test, and our neutralizing tests are well correlated and can effectively identify convalescent COVID-19 (12). Therefore, these two tests can be used to quantitatively and qualitatively evaluate antibodies against SARS-CoV-2.

In confronting emerging variants, it is necessary to understand the immunity acquired by the BNT162b2 vaccine. In the present study, sera collected after a mass inoculation of health care workers were systematically obtained at our institute. Using two antibody assays, we investigated the relationship of the immune status acquired by vaccination with the age of the vaccine recipients.

## RESULTS

### Antibody quantification and neutralizing activity after vaccination.

**FIG 1:**
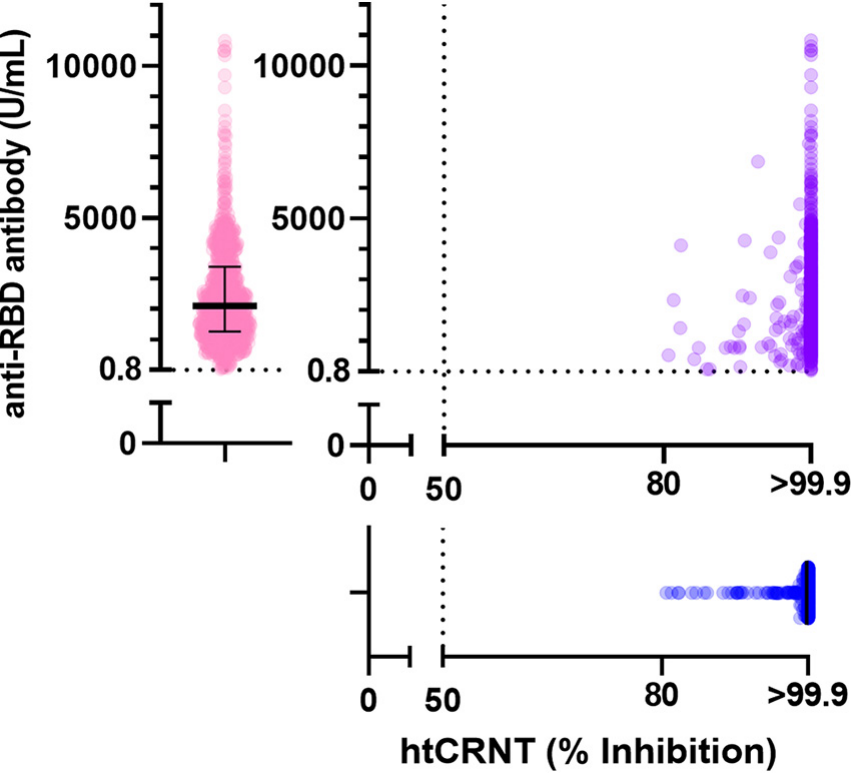
Relationship between anti-RBD antibody levels and neutralization. Neutralization levels against pseudotyped viruses measured by htCRNT (blue) and anti-RBD antibody levels measured by a commercially available test (pink) were plotted (*n* = 740). The results of both tests are plotted on the XY coordinate (purple). The value of htCRNT is defined as the mean of duplicate assays using 100-fold diluted serum. Bars indicate medians with interquartile ranges. htCRNT, high-throughput chemiluminescent reduction neutralizing test; RBD, receptor-binding domain.

Serum samples were obtained from a total of 740 participants. All participants were positive for the htCRNT and anti-RBD antibody tests (Fig. 1). All htCRNT values were over 80.0% (range, 80.6 to 100.0%), and the median was >99.9% (IQR >99.9 to >99.9%). For the anti-RBD antibody test, the range and the median were 28.5 to 10,824 U/ml and 2,112 U/ml (IQR 1,275 to 3,390 U/ml), respectively.

**FIG 2:**
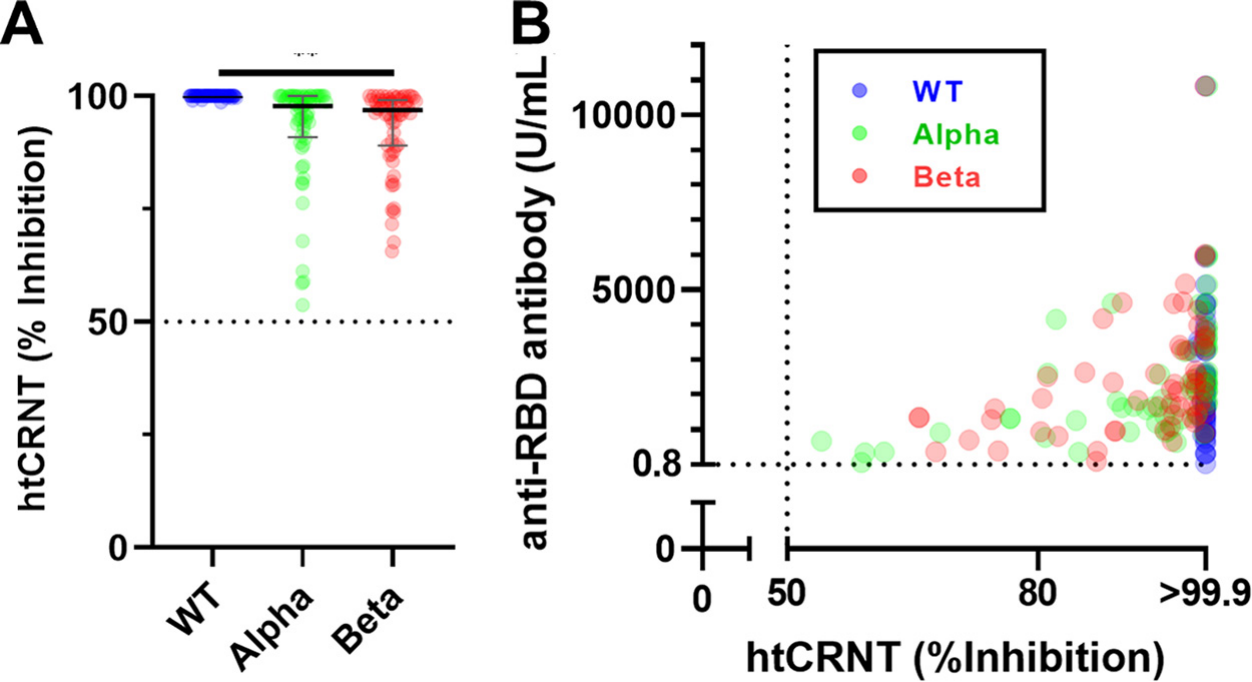
Cross-reaction against variants in representative sera. (A) Neutralizing activity against wild-type (WT), α-, and β-derived variants (*n* = 61). (B) The relationship between neutralizing activity and anti-RBD antibody levels. **, *P* < 0.01. Bars indicate medians with interquartile ranges.

Next, 61 representative samples were randomly selected and assayed using pseudotyped α- and β-derived variants. Of these, 100.0% (61/61) had positive htCRNT values against both variants. Compared to htCRNT values against the wild type (median >99.9%; IQR >99.9% to >99.9%), those against the α- and β-derived variants were significantly decreased (median 97.8%, IQR 90.9 to 99.9 and median 96.9, IQR 89.0 to 99.2, respectively; *P* < 0.01) (Fig. 2A). The htCRNT values against the α- and β-derived variants were positively correlated with the values of the anti-RBD antibody test (*r* = 0.44, 95% confidence interval [CI] 0.21 to 0.62; and *r* = 0.40, 95% CI 0.17 to 0.60, respectively) (Fig. 2B).

### Relationship between vaccine-induced antibody and demographic characteristics.

Finally, demographic characteristics were obtained from the participants who answered the questionnaire, and their test results were compared. A total of 237 participants provided their ages and their symptoms after vaccination (Table 1). Of these, 21 had data which indicated neutralization against the variants.

**TABLE 1:** Demographic and clinical characteristics of the study participants who answered the questionnaire

Profile	Answered individuals, *n* = 237
Sex, male, *n* (%)	85 (35.9)
Age, yrs, *n* (%)	
20–24	10 (4.2)
25–29	30 (12.7)
30–34	40 (16.9)
35–39	20 (8.4)
40–44	46 (19.4)
45–49	28 (11.8)
50–54	19 (8.0)
55–59	23 (9.7)
60–64	19 (8.0)
≥65	2 (0.8)
Symptoms, *n* (%)	
Local symptoms	204 (86.1)
Systemic symptoms	202 (85.2)
Underlying diseases, YES, *n* (%)	43 (18.1)
Medication, YES, *n* (%)	8 (3.4)

**FIG 3:**
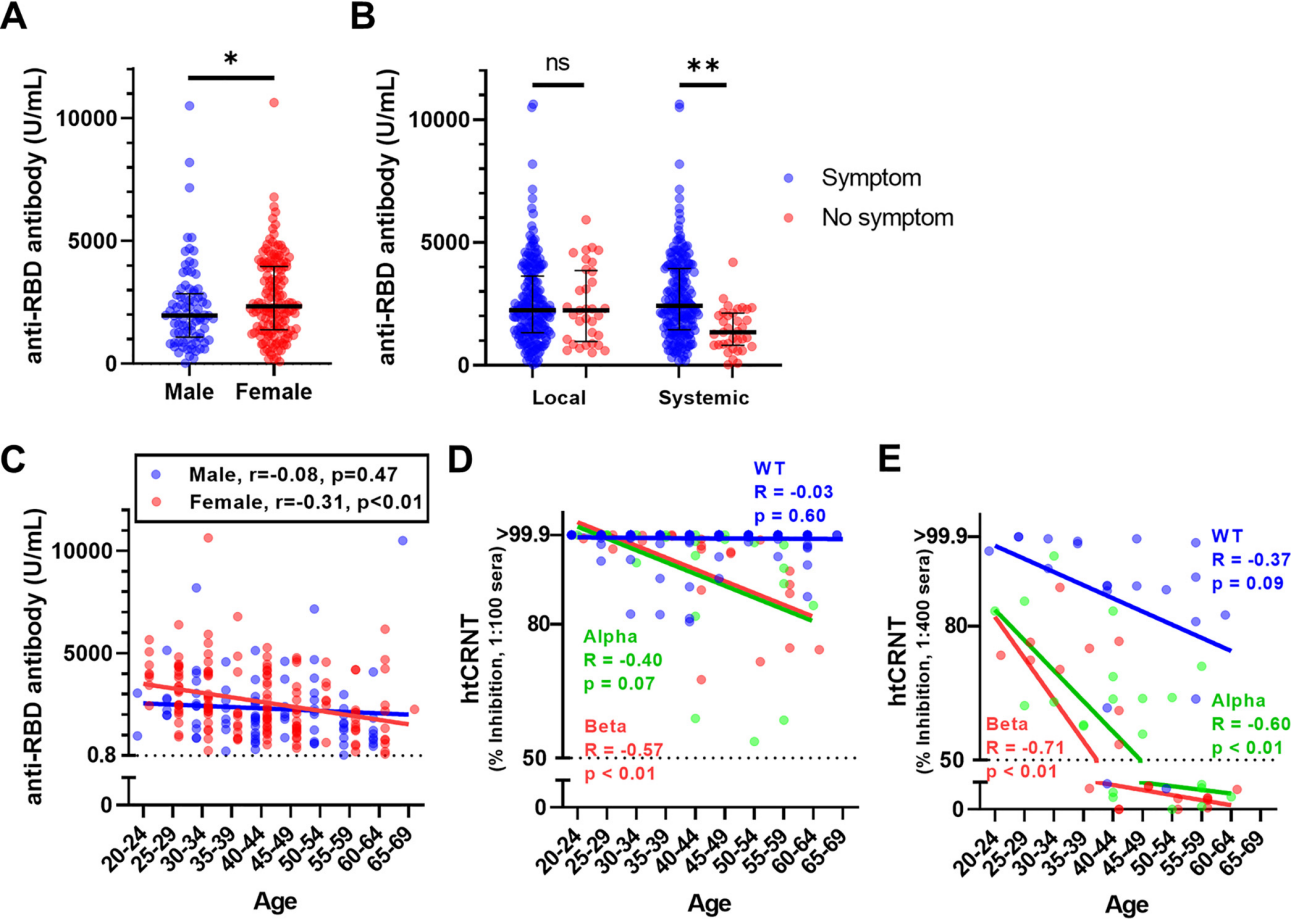
Relationship of vaccine-induced antibody levels and demographic characteristics in questionnaire-answered population. (A) Anti-RBD antibody levels in males and females (*n* = 237). (B) Anti-RBD antibody levels and local or systemic symptoms (*n* = 237). (C) Relationship between anti-RBD antibody levels and age. (D) Relationship between htCRNT levels using 100-fold dilutions of sera and age (for WT pseudotyped virus; *n* = 237, for α- and β-derived variants; *n* = 21). (E) Relationship between htCRNT levels using 400-fold dilutions of sera and age (*n* = 21). *, *P* < 0.05; **, *P* < 0.01; ns, not significant. Bars indicate medians with interquartile ranges. RBD, receptor-binding domain; htCRNT, high-throughput chemiluminescent reduction neutralizing test.

The values of the anti-RBD antibody test in females (median, 2,345; IQR 1,388 to 3,960) were significantly higher than the test values for males (median 1,967; IQR, 1,086 to 2,849; *P* < 0.05) (Fig. 3A). The systemic symptoms after vaccination were related to anti-RBD antibody levels, and the number of individuals without systemic symptoms significantly decreased compared to the number of those with systemic symptoms (with systemic symptoms, median 2,426, IQR 1,450 to 3,933; without systemic symptoms, median 1,347, IQR 818 to 2,125; *P* < 0.01) (Fig. 3B). There was no significant change in local symptoms. In individuals with underlying diseases or medications, there were no significant differences in anti-RBD antibody levels. The values of the anti-RBD antibody test were negatively correlated with age for females (*r* = −0.31, 95% CI −0.45 to −0.16; *P* < 0.01) but not for males (*r* = −0.08; 95% CI −0.29 to 0.14; *P* = 0.47) (Fig. 3C).

For neutralization, the values for htCRNT using 100-fold diluted sera against the wild-type virus were not correlated with age, while the htCRNT values for the same test against the variants decreased in an age-dependent manner (α, *P* = 0.07, *r* = −0.40, 95% CI −0.71 to 0.03; β, *P* < 0.01, *r* = −0.57, 95% CI −0.81 to 0.19) (Fig. 3D). To evaluate the relationship between vaccinee age and neutralization against the wild-type virus, htCRNT was also performed using 400-fold diluted sera. Neutralization against α- and β-derived variants, but not against the wild-type virus, showed a significant negative correlation with age (wild-type, *P* = 0.09, *r* = −0.38, 95% CI −0.70 to 0.07, *P* < 0.01; α, *r* = −0.60, 95% CI −0.82 to −0.23, *P* < 0.01; β, *r* = −0.71, 95% CI −0.87 to −0.41, *P* < 0.01) (Fig. 3E).

## DISCUSSION

Some clinically used antibody tests, such as the anti-RBD test used in the present study, quantitatively provide antibody levels as an *in vitro* diagnostic test. In clinical settings, physicians can determine acquired immunity in terms of the amount of antibody; however, this test does not provide information on variant-specific antibodies or neutralization. Therefore, measuring neutralizing activity against variants is a way to understand the function of acquired immunity.

The humoral response induced by BNT162b2 vaccine has been demonstrated by vaccine efficacy studies (1, 13–15). The present study also showed that all vaccinated individuals acquired the antibody 2 to 3 weeks after the BNT162b2 vaccination. As it has been reported that vaccinated individuals have much higher antibodies than previously-infected individuals (16), the median level of anti-RBD antibody induced by vaccination was 60.3-fold elevated, compared with our previous findings for convalescent COVID-19 (median 35.0, IQR 7.63 to 137.0) (12). Vaccinated sera also neutralized pseudotyped viral infection almost completely, while the median CRNT value for the convalescent-phase sera was 83.5 (IQR 64.1 to 90.0) (12). These findings suggest that the BNT162b2 vaccine can provide more antibodies and stronger neutralization than the immunity acquired by natural infection.

Similar to previous reports (8, 10), our findings support the notion that vaccinated sera can cross-react with SARS-CoV-2 variants. The reduction in neutralization of the β variant has been widely recognized (11, 13, 17); however, some reports have indicated that neutralization activity against the α variant is similar to that against the wild type (10, 18), and some have reported a reduced level of neutralization in the α variant compared to the wild type (8, 9, 19). In the present study, although neutralization against variants was significantly reduced compared to the wild type, almost all sera still had a >50% inhibitory effect. Approximately half of the convalescent-phase sera did not show >50% inhibition in our previous study, suggesting that vaccine-induced antibodies are likely to be more effective against variants than infection-induced antibodies for at least 2 to 3 weeks post-BNT162b2 vaccination. In addition, this increased efficacy against variants seems to be quantitatively related to the larger amount of antibody induced by the vaccine.

Sex differences were observed, with females developing higher antibody levels in vaccine-induced immunity. Similar differences in vaccination have also been reported in previous studies (20, 21). The acquisition of antibody levels was reduced in an age-dependent manner in females. This age-related reduction following the BNT162b2 vaccination was also indicated using another commercial antibody test (4, 5). These findings are consistent with a general understanding of vaccinated immunity (3). Our data also showed that the age-dependent decline in neutralization was more strongly observed against the variants than against the wild-type virus. These findings are similar to a previous report in that neutralization showed a negative correlation with age, but they differ in that the trend strongly appeared against variants (19). Because the data support the age-related immune response heterogeneity to BNT162b2 as previously reported (19, 22), older age groups may be at a higher risk of variant infection even after vaccination. Meanwhile, it remains unknown how much a reduction in neutralization will impact a patient’s future risk of COVID-19. Whether these findings in age and sex have clinical impact should be studied in the future.

The limitations of the current study are as follows. The efficacy of SARS-CoV-2 vaccination among people whose immunity may be unstable, such as children, elderly people, and individuals with underlying diseases, is unknown. It is still unknown how long immunity is maintained after vaccination. Therefore, we plan to conduct a follow-up study for the participants. Lastly, it is impossible to exclude possible participants who had already acquired immunity because sera prior to vaccination were not assessed.

In conclusion, the BNT162b2 vaccine produced anti-RBD antibodies, and neutralizing effects against both WT SARS-CoV-2 and its emerging variants, in all vaccinees. However, the neutralization against viral variants declined with increasing vaccinee age. Because variants are circulating, age-related heterogeneity of the vaccine-acquired immunity is a concern in preventive strategies.

## MATERIALS AND METHODS

### Collection of specimens.

Serum samples were collected from health care workers at the Toyama University Hospital. All patients received two doses of the Pfizer BNT162b2 vaccine, and blood samples were collected between 13 and 17 days after the second dose because patients with COVID-19 showed neutralization from 10 days after the onset of disease (12). The sera were used for serological assays either on the day of blood collection or within 3 days of storage at 4°C. The remaining sera were frozen at −80°C until further verification.

Basic clinical characteristics were arbitrarily obtained from questionnaires. The following characteristics were obtained: age, sex, local symptoms after vaccination (pain at injection site, redness, swelling, hardness, local muscle pain, feeling of warmth, itching, and others), systemic symptoms after vaccination (fever ≥37.5°C, general fatigue, headache, nasal discharge, abdominal pain, nausea, diarrhea, myalgia, joint pain, swelling of the lips and face, hives, cough, and others), underlying diseases (malignant diseases, hypertension, diabetes, dyslipidemia, renal failure, liver failure, asthma, autoimmune diseases, and others), and medications (corticosteroids excluding ointments, immunosuppressants, anti-tumor drugs, anti-rheumatoid drugs, and radiological therapy).

### Generation of pseudotyped viruses.

Pseudotyped vesicular stomatitis virus (VSV) bearing SARS-CoV-2 S protein was generated as previously described (12, 23). The expression plasmid for the truncated S protein of SARS-CoV-2, pCAG-SARS-CoV-2 S (Wuhan), was kindly provided by Shuetsu Fukushi (National Institute of Infectious Diseases, Japan). The expression plasmids for the truncated mutant S protein of SARS-CoV-2, pCAGG-pm3-SARS2-Shu-d19-B1.1.7 (α-derived variant), and pCAGG-pm3-SARS2-Shu-d19-B1.351 (β-derived variant), were constructed by PCR-based site-directed mutagenesis. S cDNA of SARS-CoV-2 was cloned into the pCAGGS-pm3 expression vector and 293T cells were transfected with the above expression vectors. After 24 h of incubation, the transfected cells were infected with G-complemented (*G) VSVΔG/Luc (*G-VSVΔG/Luc) (24) at a multiplicity of infection of 0.5. The culture supernatants containing pseudotyped VSVs were centrifuged to remove cell debris and stored at −80°C until further use.

### Serological tests.

The neutralizing effects of samples against pseudotyped viruses were examined by the htCRNT, as previously described (12). Briefly, serum diluted 100-fold with Dulbecco’s Modified Eagle Medium (DMEM, Nacalai Tesque, Inc., Kyoto, Japan) containing 10% heat-inactivated fetal bovine serum was incubated with pseudotyped SARS-CoV-2 for 1 h. After incubation, VeroE6/TMPRSS2 cells (JCRB1819) were treated with DMEM-containing serum and pseudotyped virus. The infectivity of the pseudotyped viruses was determined by measuring luciferase activity after 24 h of incubation at 37°C. The value of samples without pseudotyped virus was defined as 0% infection and 100% inhibition, while the value of samples with pseudotyped virus but without serum was defined as 100% infection and 0% inhibition. A score with ≥50% inhibition of viral infection was considered positive for neutralization.

For the commercial assay, serum samples were tested at Toyama University Hospital using the Elecsys Anti-SARS-CoV-2 S immunoassay (Roche Diagnostics GmbH, Basel, Switzerland) to quantitatively measure antibody levels against SARS-CoV-2 RBD. The manufacturer’s cutoff value (COV) was 0.8 U/ml and the minimum value was expressed as <0.4 U/ml.

### Statistical analysis.

Statistical analysis was performed using the Mann-Whitney test to compare non-parametric groups. The Friedman test with Dunn’s test was used for multiple comparisons among the three paired groups. Correlations between test findings were expressed using Pearson’s correlation coefficients. Data were analyzed using GraphPad Prism version 8.4.3 (GraphPad Software, San Diego, CA). Statistical significance between different groups was defined as *P* < 0.05. Data are expressed as medians with interquartile ranges (IQR).

### Ethics approval.

This study was performed in accordance with the Declaration of Helsinki and was approved by the ethical review board of the University of Toyama (approval no.: R2019167). Written informed consent was obtained from all participants.
